# DOA Estimation Method for Vector Hydrophones Based on Sparse Bayesian Learning

**DOI:** 10.3390/s24196439

**Published:** 2024-10-04

**Authors:** Hongyan Wang, Yanping Bai, Jing Ren, Peng Wang, Ting Xu, Wendong Zhang, Guojun Zhang

**Affiliations:** 1 School of Mathematics, North University of China, Taiyuan 030051, Chinas202308033@st.nuc.edu.cn (J.R.); wpmath@nuc.edu.cn (P.W.); xut1980@163.com (T.X.); 2 State Key Laboratory of Dynamic Testing Technology, North University of China, Taiyuan 030051, China; wdzhang@nuc.edu.cn (W.Z.); zhangguojun1977@nuc.edu.cn (G.Z.)

**Keywords:** DOA estimation, vector hydrophone, compressed sensing, sparse Bayesian learning

## Abstract

Through extensive literature review, it has been found that sparse Bayesian learning (SBL) is mainly applied to traditional scalar hydrophones and is rarely applied to vector hydrophones. This article proposes a direction of arrival (DOA) estimation method for vector hydrophones based on SBL (Vector-SBL). Firstly, vector hydrophones capture both sound pressure and particle velocity, enabling the acquisition of multidimensional sound field information. Secondly, SBL accurately reconstructs the received vector signal, addressing challenges like low signal-to-noise ratio (SNR), limited snapshots, and coherent sources. Finally, precise DOA estimation is achieved for multiple sources without prior knowledge of their number. Simulation experiments have shown that compared with the OMP, MUSIC, and CBF algorithms, the proposed method exhibits higher DOA estimation accuracy under conditions of low SNR, small snapshots, multiple sources, and coherent sources. Furthermore, it demonstrates superior resolution when dealing with closely spaced signal sources.

## 1. Introduction

With the rapid development of underwater detection technology, DOA estimation has become increasingly crucial in military reconnaissance, marine resource exploration, and underwater communication [1,2,3,4]. However, the complexity and variability of the underwater environment, along with the reduced SNR caused by noise interference, pose significant challenges to DOA estimation. In order to address the challenges of underwater signal reception, researchers are actively exploring new equipment and technologies. Among them, two-dimensional vector hydrophones have attracted much attention because of their advantages of receiving sound pressure and particle velocity information synchronously in space. Compared with the traditional sound pressure hydrophone, it uses multidimensional information to describe the sound source more comprehensively and improve the accuracy and stability of DOA estimation [5,6]. However, further study is needed to fully utilize its advantages to achieve high-precision sound source direction estimation.

Early DOA estimation algorithms primarily revolved around beamforming techniques, such as the conventional beamforming (CBF) algorithm [7], which utilizes the spatial data received by each array element instead of the traditional time domain data. However, this type of algorithm is unable to accurately distinguish the target located in a beamwidth, posing significant limitations. Subsequently, the advent of subspace algorithms has improved the resolution. Subspace algorithms include subspace decomposition algorithms and subspace fitting algorithms. Typical subspace decomposition algorithms include the multiple signal classification (MUSIC) algorithm [8,9] and the estimation of signal parameters via rotational invariance techniques (ESPRIT) algorithm [10,11], which construct “needle shaped” spatial spectral peaks based on the orthogonality between the signal subspace and the noise subspace, breaking through the Rayleigh limit problem and greatly improving the resolution of the algorithm. However, in the case of small snapshots, the accuracy of DOA estimation is limited. Spatial fitting algorithms, such as the Maximum Likelihood (ML) algorithm [12,13], can be categorized as multidimensional parameter optimization problems. The ML algorithm performs well in low SNR and small snapshot conditions, but it requires a large amount of calculation and is difficult to apply to practical engineering.

In recent years, DL Donoho, Candès E J, and Tao T et al. proposed the compressive sensing (CS) theory [14,15,16,17]. The core of this theory is that, under the premise of sparsity, signals can be reconstructed by far fewer sampling points than required via the sampling theorem. Due to the sparsity of sound source signals in underwater environments, sparse reconstruction methods are widely used for DOA estimation. After further research, scholars have applied convex optimization algorithms and greedy algorithms to signal reconstruction and achieved many new achievements. Convex optimization algorithms, such as the Least Absolute Shrinkage and Selection Operator (LASSO) algorithm [18], are global optimality algorithms that transform the $l_{0}$-norm non-convex problem into the $l_{1}$-norm convex problem to obtain the sparse solution. However, as the number of snapshots increases, the computational complexity of these algorithms will increase, and the computational efficiency will gradually decrease. Greedy algorithms, such as the orthogonal matching pursuit (OMP) algorithm [19,20], have the advantages of high computational efficiency through iteratively selecting the most matched atoms to reconstruct the signal. However, the OMP algorithm requires a preset number of incident signals, which has limitations in practical applications.

In order to improve the accuracy and robustness of DOA estimation, researchers have begun to explore sparse reconstruction methods based on Bayesian learning. SBL [21,22] incorporates sparsity constraints into the signal reconstruction process to reduce redundant information and improve the accuracy and efficiency of signal reconstruction. The signal reconstruction process is based on the maximum likelihood principle, which can effectively extract useful information from observation data, suppress noise and interference, and achieve high-resolution and accurate DOA estimation [23,24,25]. With the continuous evolution of data characteristics and application requirements, Peter Gerstoft and other scholars extended SBL to multisnapshot sparse Bayesian learning (MSBL) [26] in 2016. MSBL can better adapt to complex and variable data environments, enhancing processing performance and application effectiveness.

Therefore, this paper combines the vector hydrophone array model with the SBL method and proposes a DOA estimation method based on the Vector-SBL algorithm. This method combines the high resolution and high precision characteristics of the SBL method, as well as the characteristics of vector hydrophones to simultaneously receive sound pressure and particle information in the acoustic field, improving estimation accuracy and achieving accurate estimation of underwater sound source direction. This method does not require presetting the number of incident signals in advance for DOA estimation. Under conditions of low SNR, small snapshots, multiple sources and coherent sources, it has higher estimation accuracy and resolution and is more advantageous than traditional algorithms.

## 2. Two-Dimensional Vector Hydrophone Array Signal Model

Vector hydrophone array signal models are typically categorized into several types depending on the diverse positional arrangements of their components. In this paper, we have conducted in-depth research specifically on the uniform linear array [27]. The two-dimensional vector hydrophone array signal model only considers the horizontal azimuth angle.

**Figure 1:**
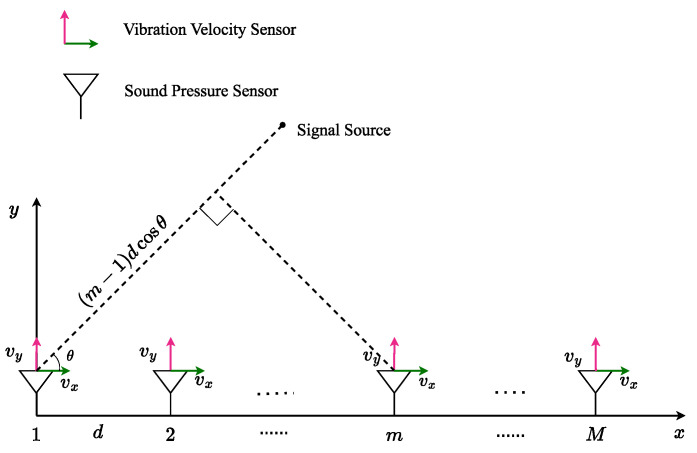
Two-dimensional vector hydrophone array signal receiving model.

The number of array elements is set to M, the array element spacing is d, the number of signal sources is K, the corresponding K narrowband far-field signals are $s_{1}(t),s_{2}(t),\cdots ,s_{K}(t)$, and the incident angle of the far-field signal is $\theta_{1},\theta_{2},\cdots ,\theta_{K}$. The two-dimensional vector hydrophone array signal receiving model is presented in Figure 1.

In Figure 1, $v_{x}$ and $v_{y}$ are the components of the vibration velocity vector $\vec{v}(t)$ along the *x*-axis and *y*-axis. At time t, there exists the following relationship between the sound pressure scalar p(t) and the vibration velocity vector $\vec{v}(t)$:

(1) $$\vec{v}(t)=[\cos\theta \cdot \vec{\xi }+\sin\theta \cdot \vec{\eta }]p(t)$$

where $\vec{\xi }$ and $\vec{\eta }$ are mutually orthogonal unit vectors.

In the two-dimensional case, the relationship between the sound pressure scalar p(t) and the vibration velocity vector component $\left\{v_{x},v_{y}\right\}$ can be expressed as follows:

(2) $$\left\{\begin{array}{l} p(t)=s(t)\\ v_{x}(t)=p(t)\cos\theta \\ v_{y}(t)=p(t)\sin\theta \end{array}\right.$$

where s(t) is the received scalar signal.

The output of a single vector hydrophone in the case of a single source is as follows:

(3) $$\begin{array}{c} x(t)=[p(t),p(t)\cos\theta ,p(t)\sin\theta ]\\ =p(t)[1,\cos\theta ,\sin\theta ]\\ =s(t)[1,\cos\theta ,\sin\theta ]. \end{array}$$

Therefore, in practical applications where the K narrowband far-field signal is $s_{1}(t),s_{2}(t),\cdots ,s_{K}(t)$, the output of a single vector hydrophone is as follows:

(4) $$x(t)=\sum_{k=1}^{K}a(\theta_{k}){\left[1,\cos\theta_{k},\sin\theta_{k}\right]}^{T}s_{k}(t)+n(t)$$

According to Figure 1, it is easy to determine that the delay between the signal source reaching the first array element and m th array element is (m−1)dcosθ, so the wave path difference is τ=(m−1)dcosθ/c (c is the speed of sound in water). So, the phase difference is $\psi =\exp(-jw_{0}\tau )=\exp(-j2\pi (m-1)d\cos\theta /\lambda )$ ($w_{0}=2\pi f_{0}$, $\lambda =c/f_{0}$ is the signal wavelength and $f_{0}$ is the signal center frequency).

Therefore, using the first element as the reference element, the output of the m th element at time t can be expressed as follows:

(5) $$x_{m}(t)=\sum_{k=1}^{K}a_{m}(\theta_{k}){\left[1,\cos\theta_{k},\sin\theta_{k}\right]}^{T}s_{k}(t)+n_{m}(t)$$

where $x_{m}(t)\in {\mathbb{C}}^{3\times 1}$; $a_{m}(\theta_{k})=\exp(-j2\pi (m-1)d\cos\theta_{k}/\lambda )$; and $n_{m}(t)\in {\mathbb{C}}^{3\times 1}$ is the noise vector received by the m th array element.

The array output X(t) is a matrix composed of M array element outputs, i.e., $X(t)={\left[x_{1}(t),x_{2}(t),\cdots ,x_{M}(t)\right]}^{T}\in {\mathbb{C}}^{3M\times 1}$. So, the matrix form of the array output can be expressed as follows:

(6) X(t)=A(θ)S(t)+N(t)

where $S(t)={\left[s_{1}(t),s_{2}(t),\cdots ,s_{K}(t)\right]}^{T}\in {\mathbb{C}}^{K\times 1}$ are receiving signals; $N(t)={\left[n_{1}(t),n_{2}(t),\cdots ,n_{M}(t)\right]}^{T}\in {\mathbb{C}}^{3M\times 1}$ is white Gaussian noise with a mean value of 0 and variance of $\sigma^{2}$; and $A(\theta )\in {\mathbb{C}}^{3M\times K}$ is signal steering vector ($\theta =\left\{\theta_{1},\theta_{2},\cdots ,\theta_{K}\right\}$), which is defined as the following:

(7) $$\begin{array}{cc} A(\theta ) & =[a(\theta_{1})⊗u_{1},a(\theta_{2})⊗u_{2},\cdots ,a(\theta_{K})⊗u_{K}]\\ & =\left[\begin{array}{l} \begin{array}{cccc} \begin{array}{c} a_{1}(\theta_{1})\\ a_{1}(\theta_{1})\cos\theta_{1}\\ a_{1}(\theta_{1})\sin\theta_{1}\\ \vdots \end{array} & \begin{array}{c} a_{1}(\theta_{2})\\ a_{1}(\theta_{2})\cos\theta_{2}\\ a_{1}(\theta_{2})\sin\theta_{2}\\ \vdots \end{array} & \begin{array}{l} \cdots \\ \cdots \\ \cdots \\ \ddots \end{array} & \begin{array}{c} a_{1}(\theta_{K})\\ a_{1}(\theta_{K})\cos\theta_{K}\\ a_{1}(\theta_{K})\sin\theta_{K}\\ \vdots \end{array} \end{array}\\ \begin{array}{cccc} \begin{array}{c} a_{M}(\theta_{1})\\ a_{M}(\theta_{1})\cos\theta_{1}\\ a_{M}(\theta_{1})\sin\theta_{1} \end{array} & \begin{array}{c} a_{M}(\theta_{2})\\ a_{M}(\theta_{2})\cos\theta_{2}\\ a_{M}(\theta_{2})\sin\theta_{2} \end{array} & \begin{array}{l} \cdots \\ \cdots \\ \cdots \end{array} & \begin{array}{c} a_{M}(\theta_{K})\\ a_{M}(\theta_{K})\cos\theta_{K}\\ a_{M}(\theta_{K})\sin\theta_{K} \end{array} \end{array} \end{array}\right] \end{array}$$

where

$$a(\theta_{k})={\left[a_{1}(\theta_{k}),a_{2}(\theta_{k}),\cdots ,a_{M}(\theta_{k})\right]}^{T}={\left[1,\exp(-j2\pi d\cos\theta_{k}/\lambda ),\cdots ,\exp(-j2\pi (M-1)d\cos\theta_{k}/\lambda )\right]}^{T}\in {\mathbb{C}}^{M\times 1}$$

⊗ represents Kronecker product.

## 3. DOA Estimation Based on Vector-SBL

### 3.1. DOA Estimation Model of Vector-SBL

In order to fully utilize the sparsity of spatial signals, it is assumed that a uniform grid partition is performed on the possible incident angles of the source within [0°,180°], the overcomplete dictionary of signal source angles is denoted by $\Theta =\{{\hat{\theta }}_{1},{\hat{\theta }}_{2},\cdots ,{\hat{\theta }}_{N}\}$ (N is the number of grid points, N≫M, M is the number of array elements), and the corresponding possible incident signal source is $\hat{S}(t)={\left[{\hat{s}}_{1}(t),{\hat{s}}_{2}(t),\cdots ,{\hat{s}}_{N}(t)\right]}^{T}$. Moreover, when the grid is dense enough, the number K of the signal sources actually incident on the hydrophone array is much smaller than the possible incident angle set, so the possible signal sources are sparse. Therefore, the DOA problem is transformed into a sparse representation problem, and the signal model can be expressed as the following:

(8) $$\bar{X}=A(\Theta )\bar{S}+\bar{N}$$

when L snapshots $\bar{X}=[\hat{X}(1),\hat{X}(2),\cdots ,\hat{X}(L)]\in {\mathbb{C}}^{3M\times L}$, ($\hat{X}(t)$ represents the t th snapshot of the K signals received by the array); $A(\Theta )=[a({\hat{\theta }}_{1})⊗{\hat{u}}_{1},a({\hat{\theta }}_{2})⊗{\hat{u}}_{2},\cdots ,a({\hat{\theta }}_{N})⊗{\hat{u}}_{N}]\in {\mathbb{C}}^{3M\times N}$ is an overcomplete dictionary matrix [28] (${\hat{u}}_{k}={\left[1,\cos{\hat{\theta }}_{k},\sin{\hat{\theta }}_{k}\right]}^{T}$); $\bar{N}=[N(1),\cdots ,N(L)]\in {\mathbb{C}}^{3M\times L}$ is the noise vector received by the array; and $\bar{S}=[\hat{S}(1),\hat{S}(2),\cdots ,\hat{S}(L)]\in {\mathbb{C}}^{N\times L}$ represents the zero-extension matrix of the incident signal. The K non-zero rows correspond to the true DOA position ${\hat{\theta }}_{k}\in \theta$, the other N−K rows are zero vectors, and the n th element of $\hat{S}(t)$ can be expressed as follows:

(9) $${\hat{s}}_{n}(t)=\left\{\begin{array}{ll} s_{n} & if{\hat{\theta }}_{n}={\hat{\theta }}_{k}\\ 0 & otherwise \end{array}\right.,n=1,\cdots ,N.$$

Due to N≫K, it can be seen that $\bar{S}$ is a row sparse matrix through analysis. The received signal $\bar{X}$ and the complete dictionary matrix A(Θ) are both known values. The DOA estimation can be obtained via recovering the sparse matrix $\bar{S}$ and identifying its non-zero row position. Consequently, the DOA estimation problem is transformed into a sparse signal reconstruction problem.

### 3.2. Vector-SBL Algorithm Principle

Based on SBL theory, all observed variables and unknown variables are initially modeled as random variables and follow a specific joint prior probability distribution. This distribution can be further refined into an independent prior distribution or a conditional distribution with parameters. In order to use Bayesian methods to handle the model represented in Equation (8), it is necessary to derive the posterior probability distribution of the unknown variable matrix $\bar{S}$ based on the likelihood function and prior model. This process combines the likelihood of observed data with the prior knowledge of the unknown variables and ultimately obtains the posterior distribution of $\bar{S}$ through Bayesian computation, providing the optimal estimation of unknown variables under the given observation data.

#### 3.2.1. Likelihood Function and Prior Model

Assuming the noise is Gaussian white noise with a mean value of 0 and a variance of $\sigma^{2}$, according to the combination of the linear relationship of $A(\Theta )\bar{S}$ and the superposition of Gaussian distributions in Formula (8), it can be inferred that $p(\bar{X}\;|\;\bar{S};\sigma^{2})$ is a Gaussian random process.

Therefore, for each pair of $\left\{\hat{X}(t),\hat{S}(t)\right\}$, the likelihood function of the array output can be expressed as a Gaussian distribution with a mean value of $A(\Theta )\hat{S}(t)$ and a variance of $\sigma^{2}$:

(10) $$p(\hat{X}(t)|\hat{S}(t);\sigma^{2})={\left(\pi \sigma^{2}\right)}^{-M}\exp(-1/\sigma^{2}{\left\|\hat{X}(t)-A(\Theta )\hat{S}(t)\right\|}_{2}^{2}).$$

Due to the independence among the L snapshots, there is the following:

(11) $$p(\bar{X}|\bar{S};\sigma^{2})=\prod_{t=1}^{L}p(\hat{X}(t)\;|\;\hat{S}(t);\sigma^{2}).$$

For the prior model, it is assumed that the signal sources are mutually independent and follow a Gaussian distribution with a mean value of 0. Furthermore, $\hat{S}$ is assumed to be mutually independent across L snapshots:

(12) $$p(\bar{S};\alpha )=\prod_{t=1}^{L}CN(\hat{S}(t)|0,\Lambda )$$

where $\alpha =[\alpha_{1},\alpha_{2},\cdots ,\alpha_{N}]$ is the variance vector; $\alpha_{i}(i=1,2,\cdots ,N)$ is the variance of the i th line in $\bar{S}$; and Λ=diag(α).

Variance describes the dispersion of data, in which a case a smaller variance indicates that most elements in the row are close to 0. Consequently, the hyperparameter α controls the sparsity of model $\bar{S}$. The zero rows of $\bar{S}$ correspond to non-true DOA locations, which indicates that a source amplitude of 0 (i.e., $\alpha_{i}=0$) corresponds to a non-true DOA location. Therefore, the amplitude of the estimated source $\bar{S}$ is converted to the amplitude of the estimated hyperparameter α to reduce the number of parameters to be estimated.

#### 3.2.2. Posterior Distribution

By combining the above likelihood function and prior model, Bayesian theorem can be used to derive the posterior distribution of $\bar{S}$:

(13) $$p(\bar{S}|\bar{X};\alpha ,\sigma^{2})=p(\bar{X}|\bar{S};\sigma^{2})p(\bar{S};\alpha )/p(\bar{X};\alpha ,\sigma^{2})$$

where α and $\sigma^{2}$ are hyperparameters. The evidence factor $p(\bar{X};\alpha ,\sigma^{2})$ represents the marginal distribution of the data. When hyperparameters α and $\sigma^{2}$ are specified, $p(\bar{X};\alpha ,\sigma^{2})$ becomes a negligible normalized evidence factor.

(14) $$\begin{array}{c} p(\bar{S}|\bar{X};\alpha ,\sigma^{2})\propto p(\bar{X}|\bar{S};\sigma^{2})p(\bar{S};\alpha )\\ \propto e^{-tr\left({\left(\bar{S}-\mu_{\bar{S}}\right)}^{H}\Sigma_{\hat{S}}^{-1}(\bar{S}-\mu_{\bar{S}})\right)/{\left(\pi^{N}\det\Sigma_{\hat{S}}\right)}^{L}}=CN(\mu_{\bar{S}},\Sigma_{\hat{S}}). \end{array}$$

As $p(\bar{X}|\bar{S};\sigma^{2})$ and $p(\bar{S};\alpha )$ in (11) and (12) are Gaussian distributions, their product $p(\bar{X}|\bar{S};\sigma^{2})p(\bar{S};\alpha )$ is Gaussian with posterior mean $\mu_{\bar{S}}$ and variance $\Sigma_{\hat{S}}$.

(15) $$\mu_{\bar{S}}=E\left\{\bar{S}|\bar{X};\alpha ,\sigma^{2}\right\}=\Lambda A(\Theta {)}^{H}\Sigma_{\hat{X}}^{-1}\bar{X},$$

(16) $$\Sigma_{\hat{S}}=E\left\{(\hat{S}(t)-\mu_{\hat{S}(t)}){\left(\hat{S}(t)-\mu_{\hat{S}(t)}\right)}^{H}|\bar{X};\alpha ,\sigma^{2}\right\}={\left(1/\sigma^{2}A{\left(\Theta \right)}^{H}A(\Theta )+\Lambda^{-1}\right)}^{-1}.$$

$\Sigma_{\hat{X}}$ is the covariance matrix of the array output.

(17) $$\Sigma_{\hat{X}}=E\left\{\hat{X}(t)\hat{X}{\left(t\right)}^{H}\right\}=\sigma^{2}I_{3M}+A(\Theta )\Lambda A{\left(\Theta \right)}^{H},$$

(18) $$\Sigma_{\hat{X}}^{-1}=\sigma^{-2}I_{3M}-\sigma^{-2}A(\Theta ){\left(\sigma^{-2}A{\left(\Theta \right)}^{H}A(\Theta )+\Lambda^{-1}\right)}^{-1}A{\left(\Theta \right)}^{H}\sigma^{-2}.$$

Given the known values of α and $\sigma^{2}$, the maximum a posteriori (MAP) [29] probability estimate for ${\hat{S}}_{MAP}$ is the mean of the posterior distribution $\mu_{\bar{S}}$.

(19) $${\hat{S}}_{MAP}=\mu_{\bar{S}}=\Lambda A{\left(\Theta \right)}^{H}\Sigma_{\hat{X}}^{-1}\bar{X}.$$

From this, it can be intuitively understood that Λ controls the row sparsity of ${\hat{S}}_{MAP}$; specifically, when $\alpha_{i}=0$, the i th row of ${\hat{S}}_{MAP}$ is $0^{T}$.

#### 3.2.3. Hyperparameter Estimation

The evidence factor $p(\bar{X};\alpha ,\sigma^{2})$ is obtained via convolving the likelihood function (11) and the prior model (12).

(20) $$p(\bar{X};\alpha ,\sigma^{2})=\int p(\bar{X}|\bar{S};\sigma^{2})p(\bar{S};\alpha )d\bar{S}=\exp\left\{-tr\left({\bar{X}}^{H}\Sigma_{\hat{X}}^{-1}\bar{X}\right)\right\}/{\left(\pi^{N}\det\Sigma_{\hat{X}}\right)}^{L}.$$

Taking the log-likelihood function and maximizing it:

(21) $$\begin{array}{ccc} & \underset{\alpha ,\sigma^{2}}{\max}\left\{p(\bar{X};\alpha ,\sigma^{2})\right\} & \\ = & \underset{\alpha ,\sigma^{2}}{\max}\left\{\ln p(\bar{X};\alpha ,\sigma^{2})\right\}\\ = & \underset{\alpha ,\sigma^{2}}{\max}\left\{-tr\left({\bar{X}}^{H}\Sigma_{\hat{X}}^{-1}\bar{X}\right)-L\ln(\det\Sigma_{\hat{X}})-NL\ln\pi \right\}\\ = & \underset{\alpha ,\sigma^{2}}{\max}\left\{-tr\left({\bar{X}}^{H}\Sigma_{\hat{X}}^{-1}\bar{X}\right)-L\ln(\det\Sigma_{\hat{X}})\right\}. \end{array}$$

The estimates of hyperparameters α and $\sigma^{2}$ are obtained from Equation (21).

MSBL optimizes model performance using fast factor maximization to estimate hyperparameters [26]. The goal of fast factor maximization is to estimate hyperparameters via maximizing the evidence factor, which is achieved through alternately updating hyperparameters. During the alternating update process, other hyperparameters remain fixed, and only the current hyperparameter is updated. This alternating update strategy not only simplifies the calculations but also enhances the convergence speed of the algorithm, and its local convergence has been proven [25].

The alternating update formula for fast factor maximization is expressed as follows:

(22) $$\alpha_{n}^{j+1}=\left(\alpha_{n}^{j}/\sqrt{L}\right)\cdot \left({\left\|{\bar{X}}^{H}\Sigma_{\hat{X}}^{-1}a_{n}\right\|}_{2}/\sqrt{a_{n}^{H}M_{\bar{X}}^{-1}a_{n}}\right),$$

(23) $${\left(\sigma^{2}\right)}^{j+1}=tr\left((I_{3M}-A{\left(\Theta \right)}_{M}A{\left(\Theta \right)}_{M}^{+})M_{\bar{X}}\right)/\left(N-K\right).$$

where he superscript j represents the iteration number; $A{\left(\Theta \right)}_{M}$ consists of the columns of A(Θ), with the column positions corresponding to the positions of the τ largest columns in α. When the number of signal sources K is known, τ=K is used, otherwise, τ randomly selects an integer between 0∼M. In this case, the estimation performance is still superior to the expectation maximization method [30], and different values of τ only affect the convergence of the algorithm [26]. $A{\left(\Theta \right)}_{M}^{+}={\left(A{\left(\Theta \right)}_{M}^{H}A{\left(\Theta \right)}_{M}\right)}^{-1}A{\left(\Theta \right)}_{M}^{H}$, $M_{\bar{X}}=\bar{X}{\bar{X}}^{H}/L$.

The termination condition for the iterative alternating update process is defined as follows:

(24) $$\varepsilon ={\left\|\alpha^{j+1}-\alpha^{j}\right\|}_{1}/{\left\|\alpha^{j}\right\|}_{1}\leq \gamma .$$

where γ is the threshold value and ${\left\|\cdot \right\|}_{1}$ is the $l_{1}$-norm.

Based on the aforementioned process, the flowchart of the Vector-SBL algorithm is presented in Algorithm 1 [31].

**Algorithm 1:** Vector-SBL algorithm.
(1)

$\mathrm{Input}:\;\hat{X}.$



(2)

$\mathrm{Initialization}:\alpha ,\;\sigma^{2},\;j_{\max}\;(\mathrm{Max Iterations}),\;\gamma .$



(3)

$\mathrm{While}\;(\varepsilon <\gamma )\;\mathrm{and}\;(j<j_{\max}),$


  (i)  
$\mathrm{Calculate}\;\Sigma_{\hat{X}}=E\left\{\hat{X}(t)\hat{X}{\left(t\right)}^{H}\right\}=\sigma^{2}I_{3M}+A(\Theta )\Lambda A{\left(\Theta \right)}^{H}.$
  (ii)  
$\mathrm{Update}\alpha \mathrm{and}\;\sigma^{2}\;\mathrm{using Equations (22) and (23).}$
  End while.
(4)

$\mathrm{Output}\alpha ,\;\sigma^{2}.$



(5)

Perform spectral peak search on the hyperparameter α to obtain the DOA estimation values.




## 4. Simulation and Experimentation

In this section, the performance of the proposed algorithm was analyzed via simulation experiments. The OMP, MUSIC, and CBF algorithms were selected for comparison with the proposed algorithm. The experiments were divided into five parts: the first part compares the DOA estimation errors of each algorithm under varying snapshot numbers; the second part compares the DOA estimation errors of each algorithm under varying SNR levels; the third part is the resolution comparison; the fourth part compares the performance of each algorithm under coherent signal source condition; and the fifth part is the success rate comparison.

In the following simulation experiments, the signal incident angle range was [0°,180°], the grid spacing was 0.1°, the uniform linear array with half-wavelength spacing was employed, the number of vector uniform linear array elements was 30, the array element spacing was 0.5 m, and the noise involved was Gaussian white noise.

The first, second, third, and fifth parts were non-coherent source experiments. Therefore, under the condition of a single source, the frequency was 1000 Hz; for three single sources, the frequencies were 1000 Hz, 1200 Hz, and 1400 Hz; and for five single sources, the frequencies were 1000 Hz, 1200 Hz, 1400 Hz, 1600 Hz, and 1800 Hz. The initialization parameters and convergence parameters of Vector-SBL were α=1, $\sigma^{2}=10^{-3}$, $\gamma =10^{-8}$, and $j_{\max}=2000$.

The formula for calculating the root mean square error (RMSE) in DOA estimation is expressed as the following:

(25) $$RMSE=\sqrt{1/KP\sum_{k=1}^{K}\sum_{r=1}^{P}{\left({\hat{\theta }}_{k}(r)-\theta_{k}\right)}^{2}}.$$

where K is the number of signal sources; P is the number of Monte Carlo Simulation; ${\hat{\theta }}_{k}(r)$ is the DOA estimation of the kth source in the rth Monte Carlo Simulation; and $\theta_{k}$ is the true incident angle of the kth signal source.

### 4.1. RMSE Comparison under Varying Snapshot Numbers

**Figure 2:**
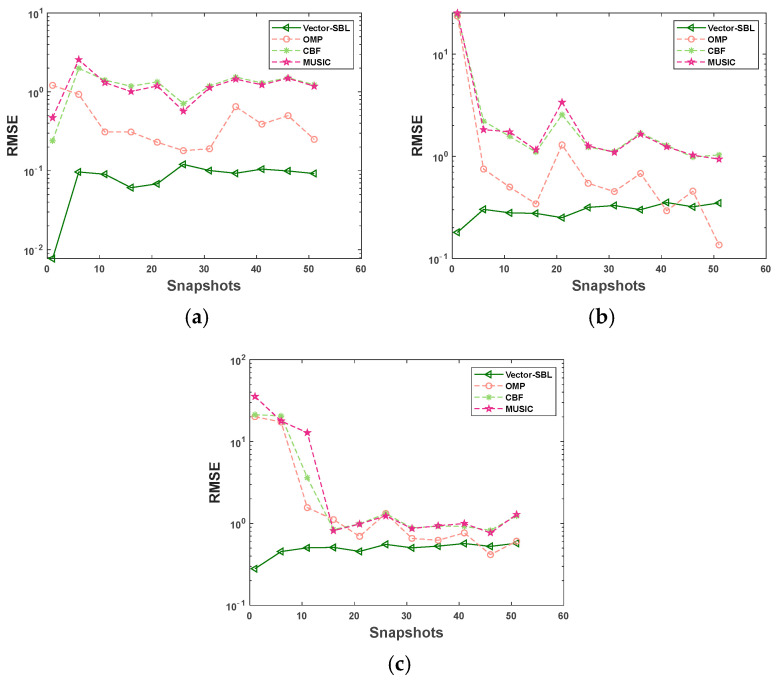
RMSE variation curves with the number of snapshots under different signal source conditions. (**a**) For 1 signal source, (**b**) 3 signal sources, and (**c**) 5 signal sources.

This experiment was conducted under a SNR of −10 dB, with the number of snapshots increasing from 1 to 51 in steps of five. For each snapshot number, 50 Monte Carlo simulations were performed. Figure 2 shows the RMSE variation curves of the Vector-SBL algorithm, OMP algorithm, CBF algorithm, and MUSIC algorithm in DOA estimation with an increasing number of snapshots under one, three, and five signal source conditions. The incident angles were set as follows: 60° for the case of one signal source, 10°, 60°, and 130° for the case of three signal sources, 10°, 30°, 60°, 80°, and 130° for the case of five signal sources. The experimental results are shown in Figure 2.

As shown in Figure 2, the performance of the Vector-SBL algorithm is significantly better than the other three algorithms with an increase in snapshots, regardless of whether in single or multiple source scenarios. It is particularly noteworthy that the estimation accuracy of the Vector-SBL algorithm was less affected by the number of snapshots. Specifically, in the case of multiple snapshots, when the number of sources was one, three, and five, the RMSR stabilized within the ranges of 0.091 ± 0.030, 0.302 ± 0.051, and 0.513 ± 0.059, respectively. This shows that the Vector-SBL algorithm can maintain a relatively stable estimation accuracy even when the number of sources increases. In addition, under extreme conditions with five sources and six snapshots, the RMSE value of the Vector-SBL algorithm had reached 0.454, whereas the RMSE values of OMP, CBF, and MUSIC algorithms were only 17.351, 20.489, and 17.798, respectively. This result highlights the superiority of the Vector-SBL algorithm under small snapshot conditions.

The exceptional performance of the Vector-SBL algorithm under small snapshot conditions was primarily attributed to its adaptive adjustment capability of hyperparameters. These hyperparameters are capable of being optimized based on different signal environments and conditions, thus ensuring that the algorithm achieves optimal estimation performance across various scenarios. Furthermore, the Vector-SBL algorithm is capable of effectively suppressing noise interference to a certain extent, providing accurate and reliable DOA estimation results for limited observational data.

In summary, in response to the problem of insufficient DOA estimation accuracy of traditional algorithms under small snapshot conditions, the Vector-SBL algorithm provides strong technical support for practical applications.

### 4.2. RMSE Comparison under Varying SNR

**Figure 3:**
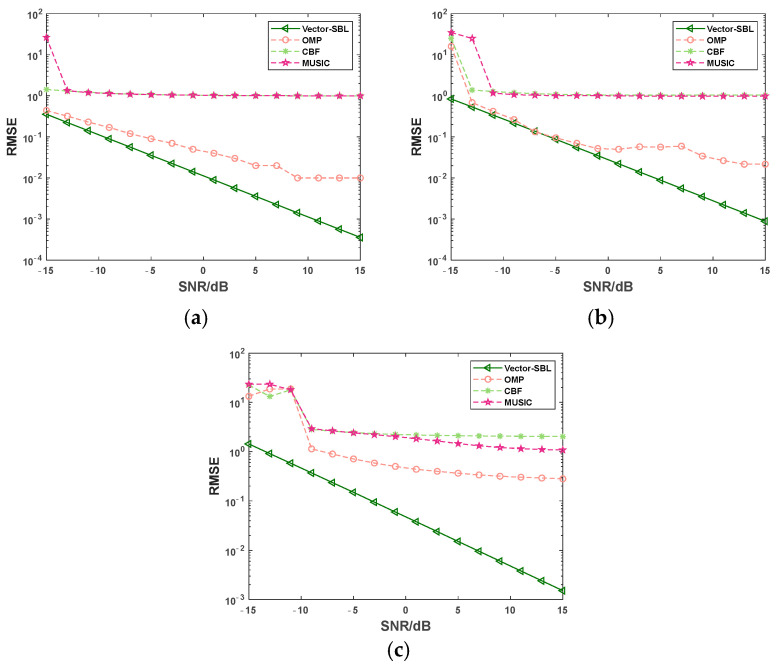
RMSE variation curves with SNR under different signal source conditions. (**a**) For 1 signal source, (**b**) 3 signal sources, and (**c**) 5 signal sources.

This experiment was conducted with a fixed number of snapshots set to 10, and the SNR was gradually increased from −15 dB to 15 dB in steps of 2 dB. 50 Monte Carlo simulations were performed for each SNR condition. Figure 3 shows the variation curves of the RMSE in DOA estimation as a function of SNR for the Vector-SBL algorithm, OMP algorithm, CBF algorithm, and MUSIC algorithm under the conditions of one, three and five signal sources. The incident angles were set as 60° in the case of one signal source, 10°, 60°, 130° in the case of three signal sources, and 10°, 30°, 60°, 80°, 130° in the case of five signal sources. The experimental results are shown in Figure 3.

As depicted in the experimental results shown in Figure 3, regardless of the presence of a single or multiple signal sources, the RMSE of the four algorithms exhibited a significant decreasing trend as the SNR gradually increased. Furthermore, in the entire experimental environment with varying SNR, the Vector-SBL algorithm had significant advantages. It is particularly noteworthy that under extreme conditions, i.e., when there were five signal sources and the SNR was −15 dB, the RMSE value of the Vector-SBL algorithm was only 1.436, while the RMSE values of the OMP algorithm, CBF algorithm, and MUSIC algorithm were as high as 13.313, 22.580, and 23.343, respectively. This result fully demonstrates the excellent performance of the Vector-SBL algorithm under low SNR and multiple source conditions.

This advantage was mainly attributed to the Vector-SBL algorithm’s ability to select useful features for DOA estimation, effectively filtering out noise and other irrelevant factors, which enabled the Vector-SBL algorithm to maintain high estimation accuracy under low SNR conditions. In addition, the Vector-SBL algorithm models understands the structure and features of signals via selecting a few key features, making the algorithm pay more attention to the essential features of signals, thus improving the accuracy of DOA estimation.

In summary, the experimental results fully validate the superiority of the Vector-SBL algorithm over other algorithms, and especially under low SNR and multiple signal sources conditions, its performance is particularly outstanding.

### 4.3. Resolution

**Figure 4:**
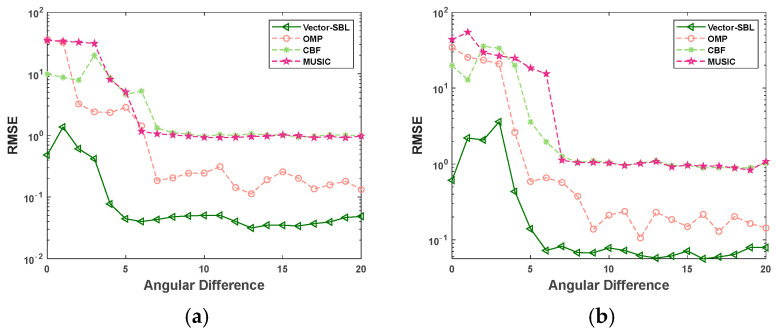
RMSE variation curves with angular difference under different signal source conditions. (**a**) For 3 signal sources and (**b**) 5 signal sources.

This experiment was conducted using a fixed number of snapshots of 10 and a fixed SNR of 0 dB. The angular difference between adjacent signal sources was gradually increased from 1 to 20 in steps of one. For each angular difference, 50 Monte Carlo simulations were conducted. Figure 4 shows the variation curves of the RMSE in DOA estimation with the angular difference for the Vector-SBL algorithm, OMP algorithm, CBF algorithm, and MUSIC algorithm under the conditions of three and five signal sources. The incident angles were set to 30°, (30 + s)°, (30 + 2 s)° for the case of three signal sources, and 30°, (30 + s)°, (30 + 2 s)°, (30 + 3 s)°, (30 + 4 s)° for the case of five signal sources. The experimental results are shown in Figure 4.

According to the experimental results shown in Figure 4, as the angular difference between adjacent signal sources gradually increased, the RMSE of all four algorithms showed a significant downward trend. Throughout the entire process of angle difference variation, the Vector-SBL algorithm consistently demonstrated significant advantages. Specifically, when the number of signal sources was three and five, respectively, the RMSE values of the Vector-SBL algorithm remained relatively stable at 0.041 ± 0.009 and 0.069 ± 0.013, respectively, for angle differences greater than 6°. In comparison, when the angular difference exceeded 10°, the RMSE values of the OMP algorithm remained relatively stable at 0.212 ± 0.100 and 0.171 ± 0.065, respectively. For the MUSIC algorithm, the RMSE values stabilized at 0.991 ± 0.074 and 0.977 ± 0.145 when the angular difference was greater than 7°. Similarly, for the CBF algorithm, the RMSE values stabilized at 1.132 ± 0.191 and 1.080 ± 0.191 for angle differences greater than 7°. These data further highlight that the Vector-SBL algorithm has higher estimation accuracy in smaller angle differences.

The aforementioned advantages originate from the fact that when dealing with neighboring signals, the OMP, MUSIC, and CBF algorithms often encounter difficulties in accurately distinguishing different signal sources, leading to a decline in estimation accuracy. However, the Vector-SBL algorithm can effectively process sparse signals, suppress noise, and enhance resolution, thus providing more accurate and stable results for DOA estimation.

In summary, the Vector-SBL algorithm has significant advantages over the OMP, MUSIC, and CBF algorithms when dealing with DOA estimation problems of neighboring signals and is particularly suitable for complex signal environments that require high-precision estimation.

### 4.4. Performance Comparison under Coherent Source Conditions

**Figure 5:**
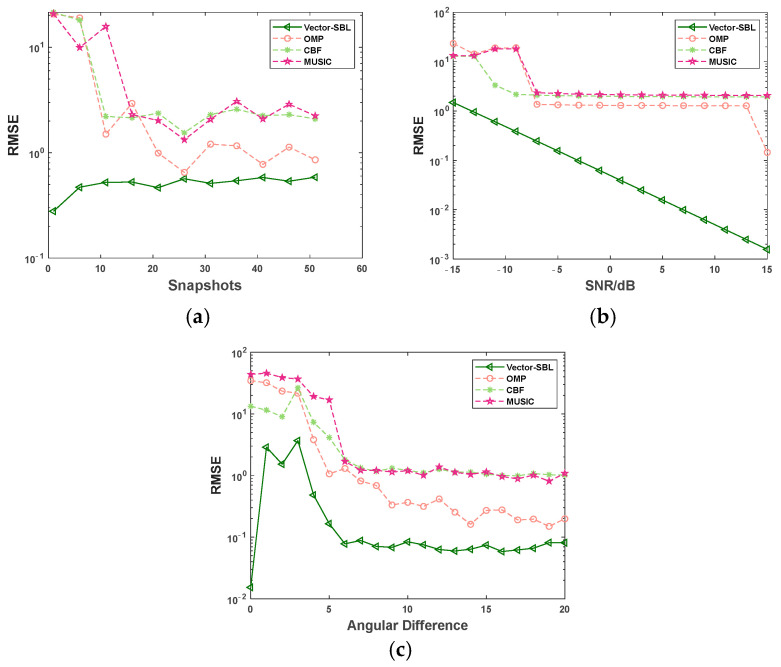
Comparison of the performance under coherent source conditions. (**a**) RMSE variation curves with the number of snapshots, (**b**) RMSE variation curves with SNR, and (**c**) RMSE variation curves with angular difference.

This experiment was conducted under the conditions of coherent signal sources, with a fixed number of signal sources set at five and a sampling frequency of 2000 Hz. While maintaining these conditions’ constants, the remaining experimental conditions were set as follows: the experimental conditions for varying snapshot numbers were conformed to those described in Section 4.1, the experimental conditions for varying SNR were conformed to those described in Section 4.2, and the experimental conditions for resolution were conformed to those described in Section 4.3. The experimental results are shown in Figure 5.

According to the experimental results in Figure 5, it was found that under all experimental conditions in this section, the Vector-SBL algorithm outperformed the comparison algorithm. Through comparing the data of the three experiments in this section with the experimental data corresponding to the same conditions in Section 4.1, Section 4.2 and Section 4.3, it was found that the maximum and minimum orders of magnitude difference for the Vector-SBL algorithm were $10^{-1}$ and $10^{-5}$, respectively. In contrast, the OMP, CBF, and MUSIC algorithms exhibited maximum and minimum difference of $10^{1}$ and $10^{-3}$ in their respective orders of magnitude. (The order of magnitude difference here refers to the difference in the order of magnitude between the coherent source RMSE and the incoherent source RMSE under the same algorithm type and experimental conditions. The maximum and minimum values of this order of magnitude difference were selected via comparing and ranking all the differences in orders of magnitude obtained from the three different experimental environments shown in Figure 5.) These data highlight the stability and accuracy of the Vector-SBL algorithm under coherent source conditions.

The reasons for this experimental result are as follows: The Vector-SBL algorithm uses prior knowledge to sparsely represent signals, accurately describing their characteristics. When processing coherent signal sources, the Vector-SBL algorithm utilizes sparsity constraints to effectively separate and reconstruct signals, improving the accuracy and stability of signal processing. The orthogonality of the signal subspace and noise subspace is disrupted in the MUSIC algorithm under coherent sources, making it unable to effectively distinguish and locate signal sources. The OMP algorithm, due to its greedy selection approach, tends to fall into local optimal solutions in the case of coherent signal sources. On the other hand, the CBF algorithm exhibits poor estimation performance for closely spaced signals due to its relatively large beamwidth.

In summary, the Vector-SBL algorithm outperforms the OMP, MUSIC, and CBF algorithms in the DOA estimation problem when dealing with coherent signal sources.

### 4.5. Success Rate

**Figure 6:**
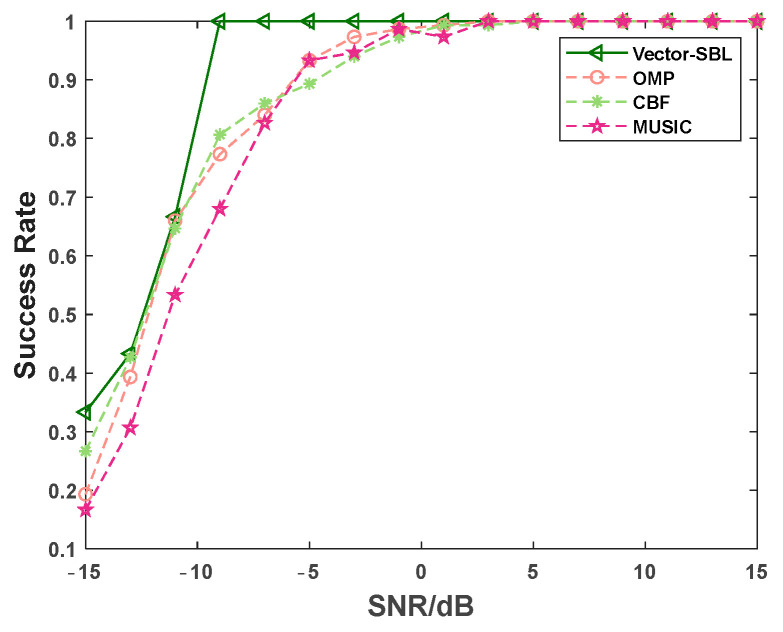
Curve of success rate versus signal-to-noise ratio.

In this experiment, the success of DOA estimation was defined as when the estimated angle was within ±0.25° of the true angle. The number of incident signal sources was set to three, with angles of 10°, 60°, and 130°, and the number of snapshots was 10. The SNR in the experiment increased from −15 dB to 15 dB with a step size of 2 dB. Fifty Monte Carlo experiments were conducted under each SNR condition. The experimental results are shown in Figure 6.

From Figure 6, it is evident that the success rates of all four algorithms increased as the SNR increased. Moreover, the proposed Vector-SBL algorithm outperformed the other three algorithms in terms of success rate at each SNR level, which fully demonstrates the ability of the Vector-SBL algorithm to accurately process signals in complex environments. Notably, under the challenging condition of an SNR of −9 dB, the Vector-SBL algorithm achieved a remarkable 100% success rate. This result indicates that the Vector-SBL algorithm can maintain high accuracy even when facing significant noise interference. In summary, the Vector-SBL algorithm proposed in this paper is superior to other algorithms.

## 5. The Lake Test Experiment of MEMS Vector Hydrophones

In order to verify the application effect of the Vector-SBL algorithm in practical engineering, in this section, the Vector-SBL algorithm was applied to the underwater acoustic direction-finding experiment of MEMS vector hydrophones. This experiment was conducted in a reservoir located in Taiyuan, Shanxi province. The reservoir can reach a water width of over 1 km, with an average depth of about 40~50 m. The water surface was stable and the area was open, making it suitable for sound field testing.

In the experiment, we used a ciliated biomimetic MEMS vector hydrophone developed by North University of China and collected underwater acoustic signals through a horizontally uniform linear array composed of five elements. These five array elements were fixed on the side of the hull of the array ship for precise direction finding of fixed-point sound sources. The spacing between the array elements was set to 0.5 m and placed at a depth of 10 m underwater. The signal transmitter was placed on another signal ship, also located at a depth of 10 m underwater, with a distance of 40 m between the two ships.

**Figure 7:**
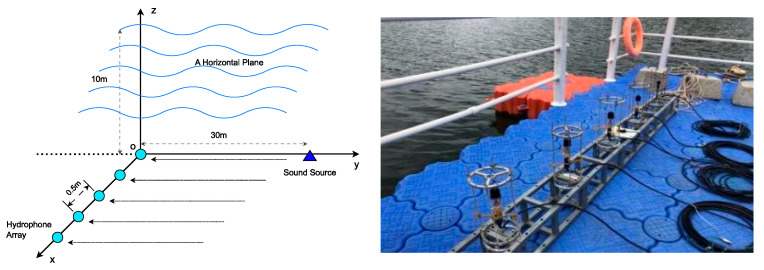
Schematic diagram of an experiment conducted at a reservoir in Taiyuan, Shanxi Province.

During the experiment, the signal transmitter located at the 90° angle of the hull of the array ship emitted a continuous sine single frequency signal with a frequency of 1 kHz. This signal was then received by the hydrophone array on the hull of the array ship for precise measurement of the sound source direction. Figure 7 shows the layout of a horizontally uniform linear array of five element MEMS vector hydrophones and the overall schematic diagram of the experiment.

**Figure 8:**
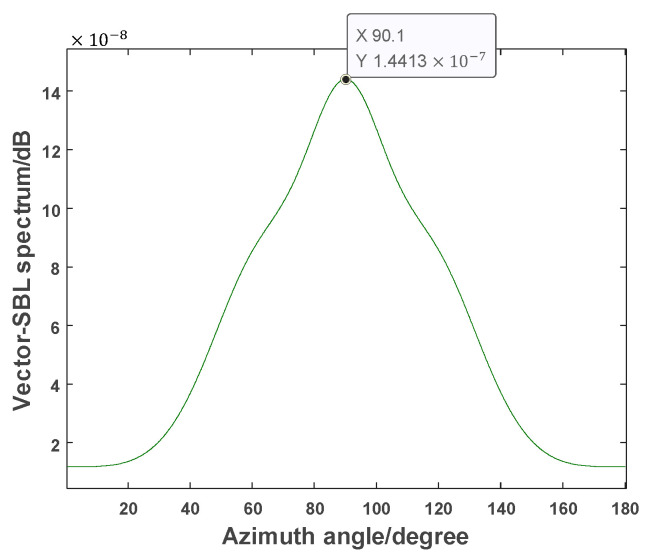
Measurement results of the Vector-SBL algorithm.

Figure 8 shows the measured results of the Vector-SBL algorithm:

From the simulation and experiments in Section 4, it can be concluded that the Vector-SBL algorithm has high estimation accuracy. The following compares the estimation performance of Vector-SBL, OMP, and MUSIC algorithms in actual testing. The measured results of Vector-SBL, OMP, and MUSIC algorithms are recorded in Table 1.

From Table 1, it can be seen that the Vector-SBL algorithm exhibits high estimation accuracy in the actual measurement environment. This significant advantage not only exists in theoretical simulations but has also been fully validated in practical applications. The Vector-SBL algorithm has shown excellent performance in both simulation experiments and real underwater acoustic environments, providing new possibilities and ideas for the application of MEMS vector hydrophones in complex underwater acoustic environments.

## 6. Conclusions

This paper proposes a DOA estimation method based on Vector-SBL to address the issues of insufficient estimation accuracy, limited resolution of similar sources, and low stability and estimation accuracy of existing vector hydrophones in the face of small snapshots, low SNR, and multiple source conditions. This method combines the signal model received by vector hydrophones with sparse Bayesian learning algorithms, achieving efficient processing of sound pressure and particle velocity information and accurate estimation of angles. Through a large number of experiments and analysis, this method shows significant advantages and characteristics. The specific summary is as follows:

(1) The Vector-SBL algorithm has higher DOA estimation accuracy compared with OMP, MUSIC, and CBF algorithms under low SNR, small snapshot, and multiple source conditions. Specifically, under extreme conditions of five signal sources, six snapshots, and an SNR of −10 dB, the Vector-SBL algorithm achieves a RMSE value of 0.454.
(2) Under coherent source conditions, the maximum order of magnitude difference in RMSE of the Vector-SBL algorithm compared with non-coherent source conditions is $10^{-1}$ while the minimum order of magnitude difference is $10^{-5}$ . This performance significantly outperforms OMP, MUSIC, and CBF algorithms, demonstrating the high stability and estimation accuracy of the Vector-SBL algorithm in DOA estimation.
(3) Under the conditions of three sources, 10 snapshots, and an SNR of −9 dB, the Vector-SBL algorithm achieves a 100% success rate, indicating its ability to maintain high accuracy even when faced with significant noise interference.

However, given the constant variations in the underwater environment and the ongoing advancements in signal processing technology, we must persist in our research efforts to further enhance and optimize this method, thus providing more reliable and efficient solutions for practical applications.

## Figures and Tables

**Table 1:** Comparison of measured results of the three methods.

Method	DOA Estimated Value	Absolute Error	Relative Error
Vector-SBL	90.1000	0.1000	0.1111
OMP	90.3000	0.3000	0.3333
MUSIC	89.7000	0.3000	0.3333

## Data Availability

The authors do not have permission to share data.
